# Please listen

**DOI:** 10.1002/jhm.70016

**Published:** 2025-02-20

**Authors:** Courtney Burnett

**Affiliations:** ^1^ University of Minnesota School of Medicine and HealthPartners, Regions Hospital Minnesota USA

I wake, eyes fluttering open

from an anesthesia‐induced slumber

Across from me in the post‐anesthesia care unit

I hear machines beeping, coarse breathing, gasping,

wheezing and fear

A man nearby sounds like he is drowning

in his own secretions

An exacerbation of congestive heart failure

my own post‐craniotomy brain tells me

I am a physician, but today I am a patient

This is a new role for me. In many ways,

it is much harder

I hear the nurse page anesthesia overhead

Respiratory assistance needed stat, bed two

In bed three, I open my lips and whisper

*He needs diuretics, positive pressure ventilation*

I can help. Let me help

I try to get someone's attention

But I am a patient, lying alone in a bed

with a bandage on my skull and blood on my cheek

Yesterday, I worked in this hospital

now I am a patient, unable to assist

My autonomy is gone, expertise unknown

My badge removed and stuffed in a bag

with my belongings and my voice

The nurse finally sees me

*Are you in pain?* He asks

I try to say no, but my words slur and disappear

I feel vulnerable and alone,

thrust to the other side, where I am seen as patient

and nothing more

*Do all of my patients feel this way?* I wonder

I hear the team arrive to help bed two

What happened? A resident asks, panic in her voice

I am an internist. I can help him

*Please listen*.

*Diuretics, positive airway pressure, elevate his head*

*the man in bed two. That is what he needs*. I whisper again

The team sees a postoperative patient mumbling words

*Why won't they listen?*

My identity is reduced to the patient in bed three

My words go unheard

*Is this what it feels like to be a patient?*

*I must never forget this*

When my patients speak, I promise to listen

## CONFLICT OF INTEREST STATEMENT

The author declares no conflicts of interest.

